# Radiation Matters of the Heart: A Mini Review

**DOI:** 10.3389/fcvm.2018.00083

**Published:** 2018-07-09

**Authors:** Kareena M. Menezes, Huichen Wang, Megumi Hada, Premkumar B. Saganti

**Affiliations:** Radiation Institute for Science and Engineering, A Texas A&M Chancellor's Research Initiative, Prairie View A&M University, Prairie View, TX, United States

**Keywords:** radiation therapy, proton therapy, heavy ion radiotherapy, ionizing radiation cardiotoxicity, charged particle therapy, cardiovascular disease, radiation damage to the heart

## Abstract

Radiation Therapy (RT) has been critical in cancer treatment regimens to date. However, it has been shown that ionizing radiation is also associated with increased risk of damage to healthy tissues. At high radiation doses, varied effects including inactivation of cells in treated tissue and associated functional impairment are seen. These range from direct damage to the heart; particularly, diffuse fibrosis of the pericardium and myocardium, adhesion of the pericardium, injury to the blood vessels and stenosis. Cardiac damage is mostly a late responding end-point, occurring anywhere between 1 and 10 years after radiation procedures. Cardiovascular disease following radiotherapy was more common with radiation treatments used before the late 1980s. Modern RT regimens with more focused radiation beams, allow tumors to be targeted more precisely and shield the heart and other healthy tissues for minimizing the radiation damage to normal cells. In this review, we discuss radiation therapeutic doses used and post-radiation damage to the heart muscle from published studies. We also emphasize the need for early detection of cardiotoxicity and the need for more cardio-protection approaches where feasible.

## Introduction

Cancer associated heart disease has become a prominent cause of mortality in the industrialized world (1). Modern treatment using radiotherapy has resulted in a dramatic improvement in the chances of cancer patient's survival. While the high energy ionized radiation treatment successfully kill cancer cells, they at the same time harm healthy cells, leading to several side effects including increased cardiovascular disease in cancer survivors (2).

It is well known that nuclear industry workers and survivors of nuclear catastrophes have a significantly higher incidence of cardiovascular diseases than the general population (3–5). For the last couple of decades, it had been found that radio therapy (RT) increases the risk of associated radiation related cardiac damage in cancer survivors (6). However, a significant increase of death rate in the follow up after 10 year was found in patients post radiation therapy (7). Later studies also revealed that radiotherapy increased the cardiovascular mortality in women treated for left breast compared to those who are treated only to the right breast from earlier studies during 1970s and 1980s (8). Several population studies show that RT induced heart disease develops very slowly and often seen around 15 years after the first exposure to radiation (9).

Subsequent studies have focused on the risk of radiation-induced heart mortality as a linear-quadratic function at moderate dose levels (10) and at high dose levels a more linear response (11–13). However, no threshold dose studies have been reported; we therefore suggest that the radiation dose exposed to the heart must be minimized and limited as there is no such thing as safe radiation dose to the heart.

Studies to-date show that radiation-associated cardiac disease emerged from studies of breast cancer (14) and Hodgkin's lymphoma (15, 16) There exists enough scientific evidence to now support radiation-related heart injury as a direct effect of RT to the chest (8) (Early Breast Cancer Trialists' Collaborative Group, EBCTCG-2000). At doses above 30 Gy, heart disease may occur within a year or two of radiation exposure with concomitant increase in the risk factors for cardiovascular disease with higher radiotherapy doses. At lower doses, the latency period is longer and can extend to more than a decade (17). Cardiovascular disease as a direct side effect of radiation was more common with radiation treatment regimens used before the late 1980s. Newer radiation protocols with lower radiation doses and more focused radiation beams allow tumors to be targeted more precisely and shield the heart and other healthy tissue from direct impact of radiation. In this review we discuss radiation induced damages to the heart tissue and effectiveness of current approaches to minimize the damage.

## Radiation induced cardiac damage

A study of radiation doses used between the 1950s and the 1990s comparing whole heart doses for left vs. right-sided breast cancer indicate that heart doses for left-sided were higher than that for the right. The dose range was shown to be 13–17 Gy for the left breast and 2–10 Gy for the right (18). Breast radiotherapy practiced in the 1970s and 1980s resulted in more exposure to the myocardium of the heart and thereby damage, which was higher when left breast was treated (Table 1). Higher cardiovascular mortality following irradiation of the left breast as opposed to the right has been attributed to this difference (19). Swedish cancer registry documents increased mortality from myocardial infarction for patients treated for left compared with right sided tumors during 1970 and 1985 (20).

**Table 1:** Relative risk of Cardiac mortality after radiation for left vs. right breast cancer laterality at 95% Confidence Interval (CMR, Cardiac Mortality Ratio).

	**CMR (Left vs. right tumor laterality)**	**CMR (Left vs. Right tumor laterality)**	**CMR (Left vs. Right tumor laterality)**
**Diagnosis**	<**10 years**	**10–14 years**	≥**15 years**
1973–1982	1.2 (1.04–1.38)	1.42 (1.11–1.82)	1.58 (1.29–1.95)
1983–1992	1.04 (0.91–1.18)	1.27 (0.99–1.63)	NA
1993–2001	0.96 (0.82–1.12)	NA	NA

A correlation exists between RT to the thoracic region and ischemic cardiac disease with older clinical trials that are perhaps no longer standards for radiation treatment care (Table 2). It is also noted that RT for Hodgkin's lymphoma and breast cancer increases the risk factor for cardiovascular disease (26). A 3–5-fold greater incidence of cardiovascular disease has been observed in patients treated for Hodgkin's lymphoma and thereafter followed for a median of 18 years (27).

**Table 2:** Selected studies with significance for heart condition post radiation treatment.

**Author-year**	**Tissue/neoplasm**	**Average dose to heart (Gy) (mean, range)**	**Heart studies (endpoints)**	**Sample size**
Cohn et al. (6)	Hodgkin's, breast, cervix, esophagus	1.5–9	Pericardial effusion/Cardiac Damage	21
Brosius et al. (21)	Hodgkin's	3–8.8	Thickened pericardia, interstitial myocardial fibrosis, fibrous thickening of mural and valvular endocardium	16
Applefeld and Wiernik (22)	Hodgkin's thorax	3–4	Constrictive or occult constrictive pericarditis, abnormal hemodynamic response, coronary artery disease, left ventricular dysfunction	48
Orzan et al. (23)	Hodgkin's, lymphoma,breast, seminoma	45–122	Aortic stenosis, regurgitation, pericardial effusion, constrictive pericarditis, mitral/tricuspid regurgitation, myocardial infarction, pericardial effusion	15
Veinot and Edwards (24)	Hodgkin's thorax	1.3–4	Pericardial fibrosis, constrictive pericarditis, endocardial fibrosis, and valvular dysfunction, non-ischemic myocardial fibrosis, obstructive coronary artery disease with myocardial ischemia, damage to the great vessels and conduction system dysfunction	27
Darby et al. (12)	Breast	4.9 (0.03–27.72)	Myocardial infarction, coronary revascularization, ischemic heart disease	2,168 (936 cases, 1,205 control)
Erven et al. (25)	Breast/chest wall	5	Decrease in cardiac strain and strain rate	75

In contrast, most of these complications are reduced significantly with recent modern radiotherapeutic approaches that are designed to minimize direct cardiac dose such as three dimensional conformal radiotherapy (3DCRT) [(28–30)] and field-in-field techniques (31). Modern advances also contain better imaging technology approaches that help minimize the radiation doses to critical organs including the exposure to the heart. Among these, image guided radiotherapy (IGRT) (32), intensity modulated radiotherapy (IMRT) (33) and stereotactic body radiotherapy (SBRT) (34) provide more efficient conformation around the tumor volume, sparing organs at risk. IMRT (35–38) and accelerated partial breast irradiation (39, 40) along with practices such as deep inspiration breath hold (DIBH) vs. free breathing reduce the mean heart dose by about 50% with mean heart doses 2–3 Gy (41–46).

**Figure 1:**
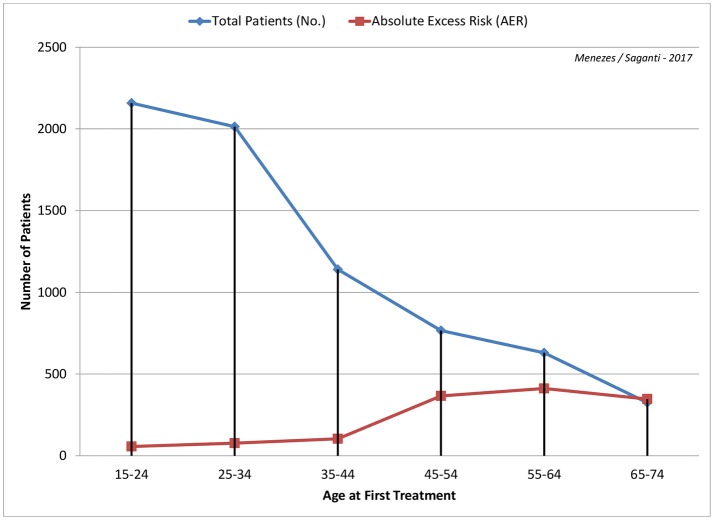
Age at first radiation treatment from 15 years through 74 years are shown with calculated Absolute Excess Risk (AER) per 1,000 patients is depicted with data from Swerdlow et al. (48). Higher the age, the greater the risk with about 50% around age 45 years and almost 100% by age 65 years.

In a study investigating the linkage of radiotherapy to cardiovascular associated deaths, the absolute risk was seen to increase within the first 10 years for coronary disease and from the next 10 for mortality (47). To this effect, earlier measurement of cardiac damage becomes crucial to better clinical therapeutic intervention (Figure 1).

A suggested protocol to identify cardiac damage - Methods that would reliably predict the progression from radiotherapy to late, irreversible cardiac damage would facilitate the development of better therapeutic measures to cardiac safety. A way to identify patients at risk for cardiac failure would help generation of some early preventive measures, individualized toward the patient. Methods could be set in place to detect and/or measure early cardiac damage such as biochemical tests. Studies for improving prediction and preventing lesions to cardiac tissue surrounding tumors such as BACCARAT (BreAst Cancer and Cardiotoxicity induced by RAdioTherapy) could improve patient care and overall quality of life (49). Atrial natriuretic peptide (ANP) levels are seen as increased in patients irradiated for Hodgkin's disease and breast cancer. This alludes to the possibility that ANP plasma levels may be an identification marker for radiation induced cardiac dysfunction (50).

## Radiation induced vascular changes

It is well documented that RT induces vascular endothelial dysfunction, which ultimately results in clinical cardiovascular events, manifesting many years after completion of therapy (51). Radiation induced heart conditions are described in selected studies (Table 3). The linkage of senescence of endothelial cells and atherosclerosis has been well established (68). In the preclinical setting, irradiation of the heart has been associated with endothelial cell dysfunction leading to accelerated atherosclerosis (69).

**Table 3:** Radiation induced heart conditions for selected studies.

**Radiation study**	**Observed condition**	**Description**
Murros and Toole (52); Stewart et al. (53)	Arteriosclerosis	Thickening of heart wall and loss of elasticity
Gujral et al. (54)	Cardiac valve diseases	Heart Valve Abnormalities
Posner et al. (55)	Cardiac arrhythmias	Irregular Heart Rate
Stewart et al. (56); McChesney et al. (57)	Cardiomyopathy	Heart muscle becomes enlarged, thick or rigid
Wright and Bresnan (58); Ivanov et al. (59); Morris et al. (60); Smith et al. (61)	Cerebrovascular disease	Lack of oxygen to brain through blood
McReynolds et al. (62); Gyenes (63); Darby et al. (12)	Ischemic heart disease	Cholesterol plaque build-up in arteries, blocking flow of blood and oxygen
Morton et al. (64); Morton et al. (65); Brosius et al. (21); Posner et al. (55); Mill et al. (66); Stewart and Fajardo (67)	Pericarditis	Inflammation of the pericardium

A more focused study with rodent models indicate that the radiation causes microvascular damage. Microvascular damage is manifested by a decrease in capillary density, resulting in chronic myocardial ischemia and fibrosis, whereas macrovascular disease is due to an accelerated onset of age-related atherosclerosis (70). Experimental data (53) lead to formulation of two possibilities for a mechanistic explanation of increased death from coronary artery dysfunction that follows exposure to radiation. The first, being radiation increases the frequency of myocardial dysfunction by affecting the biological pathway of age-related atherosclerosis. The second that radiation reduces the heart's tolerance to acute infarctions due to damage to the microvasculature, thereby increasing lethality. These two possible explanations may be contiguous and not necessarily exclusive acting together to produce heart disease.

## Charged particle therapy and heart

Particle radiation therapy applied today uses more advanced techniques and safer approaches. About 137,518 (by 2014) patients worldwide were treated with particle therapy between 1954 and 2014, 86% of which were treated with protons and 14% with carbon ions and with other particles (71). Between 2014 and 2016, in just 2 years, the total number of patients treated with particle therapy increased by 27% or 36,994 new patients to a total of 174,512 (by 2016), about 27% increase. This includes a 37% increase in new carbon ion therapy patients from 15,736 (in 2014) to 21,580 (in 2016) by 5,844. On the other hand, proton therapy patients were increased by about 26% from 118,195 (in 2014) to 149,345 by about 31,150 patients worldwide. This is a significant increase in the total number of patients who are treated with more precise radiation treatment options. A study for the late effects of radiotoxicity to the heart from this new class of patient database after 5 and 10 years is of great importance for detailed studies and assessment. Such studies are anticipated and expected to dominate the published literature in the next few years. More details of the ion therapy data worldwide are shown in Figure 3 for protons and carbon ions and in Table 4 for all other particle therapy patients.

**Table 4:** Total number of patients who received treatment with protons, carbon, pion, helium, and other ions around the world through 2017.

**Country**	**Protons**	**Carbon**	**Pion**	**Helium**	**Other**	**All**
Belgium	21					21
Canada	196		367			563
China	1,239	563				1,802
Czech Rep.	1,538					1,538
England	3,020					3,020
France	13,903					13,903
Germany	8,556	2,870				11,426
Italy	846	816				1,662
Japan	23,842	17,331				41,173
Poland	167					167
Russia	7,061					7,061
South	2,799					2,799
Sweden	1,716					1,716
Switzerland	8,106		503			8,609
Taiwan	439					439
USA	75,896		230	2,054	433	78,613
Grand total	149,345	21,580	1,100	2,054	433	174,512

*Data is adopted from PTCOG, Particle Therapy Co-Operative Group (https://www.ptcog.ch/)*.

Adjuvant breast radiotherapy dramatically reduced radiation dose to the heart and substantially decreased the risk of death from cardiovascular heart disease (72, 73). More efficient planning with CT scanners and accurate delivery with IMRT could be ways to protect the heart and lungs from unintentional radiation (74).

Radiation treatment with x-rays and gamma particles, which emit high energy electromagnetic radiation is absorbed completely into the target tissue, resulting in an increase of radiation dose per tissue depth. Proton and heavy ions such as carbon ions which constitute charged particles, deposit minimal energy at the entrance of the body where their velocity is greater and deposit most of the energy at the end of its range (as planned and calculated for the Bragg peak) in the tumor. Charged particles therefore present a newer advancement to RT to achieve lower and more targeted dose to tumor and reduce organ at risk (OAR). Since cardiac damage is a late event, long term follow-up data to study its effects on the heart are limiting. Charged particles operate by delivering high energy more effectively than x-rays or gamma particles, therefore they have an advantage of exhibiting a higher control of the tumor, lower probability of damage to healthy tissue, low risk of complications and a good prognosis for a rapid recovery after therapy (75); thus it is most promising for cardio-protection than conventional radio therapy.

Proton therapy may spare radiation exposure to the heart and reduce cardiotoxicity (18). The main benefit of proton therapy in breast cancer is to spare the heart from direct radiation exposure (76). The heart dose is dramatically reduced in proton therapy. A study on left breast cancer treatment using intensity radiotherapy and proton therapy using normal tissue probability showed that proton therapy has less radiation dose and damage to the heart (77). However, whether the cardiovascular disease is reduced in breast cancer survivors from proton therapy remains unclear. An undergoing study will reveal whether proton therapy decreases radiation induced cardiovascular disease in breast cancers.

**Figure 2:**
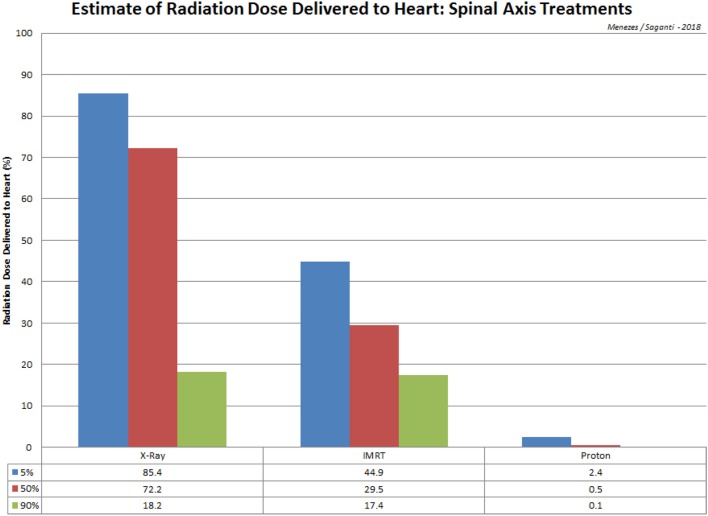
A comparison of radiation treatment via spinal axis and the estimated dose received at the heart for X-Ray, IMRT, and Proton procedures. Data is adopted from St Clair et al. (80).

In addition to the advantage of proton therapy, carbon therapy delivers higher linear energy transfer radiation (LET). High LET radiation increases radiation sensitivity to radioresistant cancer and overcomes the oxygen enhancement ratio (OER). Carbon ion therapy has also been used to stage I breast cancer without surgery at National Institute of Radiological Science (NIRS; Chiba, Japan) (78). Significant sparing of normal tissue has been demonstrated with IMRT (Intensity-modulated radiation therapy) proton treatment (79, 80), such that the dose delivered to 90% of the cochlea was reduced from 101.2% with conventional x-rays to 33.4% for IMRT beams and 2.4% for proton beams. Dose calculations to the heart recorded a reduction from 72.2% with conventional x-rays to 29.5% with IMRT and merely 0.5% with protons (Figure 2).

**Figure 3:**
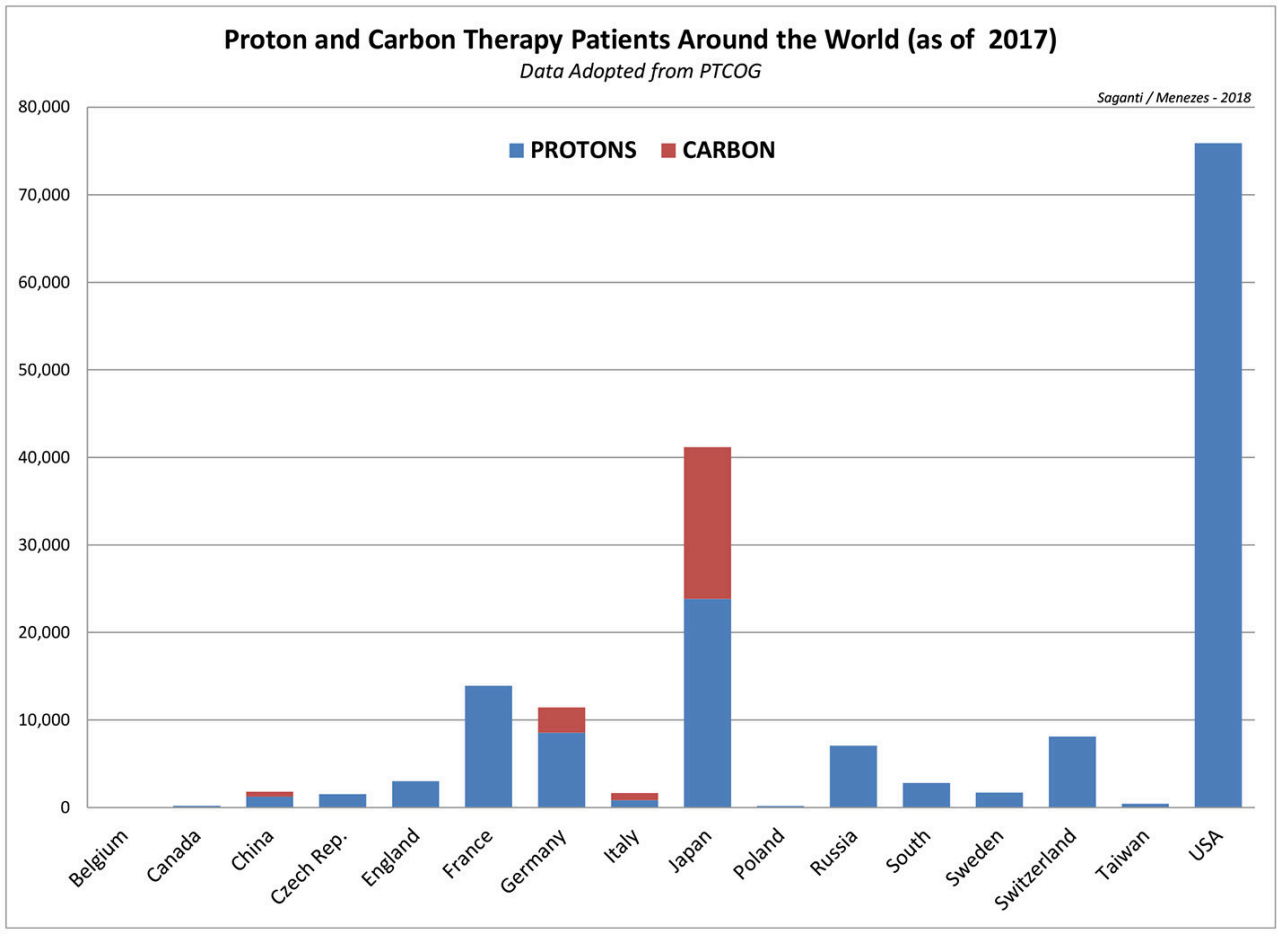
Depiction of worldwide patients treated with protons and carbon ions as of 2017 indicating largest number patients treated with protons (75,896) in the US and patients treated with carbon ions (17,331) in Japan. Data is adopted from PTCOG, Particle Therapy Co-Operative Group (https://www.ptcog.ch/).

## Non radiation approaches for preventing damage to the heart

Just as any other decease-prevention, mitigation, and treatment of radiation-induced cardiac injury also demands early detection. The sequences of events leading to cardiac damage that result from radiation are of several facets. To identify an early detection marker to predict risk of radiation induced cardiovascular disease is a key to prevent the late effects. Ionizing radiation induce premature aging in cultured endothelial cells (ECs) can be seen as increased apoptosis and expression of inflammatory markers (81) which *in vivo* are associated with EC dysfunction and atherosclerotic plaque formation (82). It has been also reported that the biological effects of ionizing radiation exposure activate NF-κB, and reduces anti-inflammatory gene expression, which *in vivo* are pro-atherogenic conditions (83). Also, p90RSK is a unique serine/threonine kinase with two distinct functional kinase domains (84) that has been well characterized for its role in heart failure (85, 86). Perhaps, phenomena can be used as an early detection marker of radiation induced late cardiovascular diseases.

Pharmaceutical approach to prevent RT-related cardiac injury - since the endothelium of the vasculature is thought to be one target for injury induced by radiation, pharmaceutical interventions to maintain endothelial functions are one potential strategy to mitigate and treat radiation-induced cardiac damage. The pharmaceutical drug *Captopril*, which is currently used to treat hypertension and congestive heart failure because of its function as angiotensin-converting enzyme (ACE) inhibitor, has been known to be able to prevent structural changes to the heart, when administered after radiation exposure (20 Gy), but there is no evidence seen in its ability to prevent the decline in cardiac function (87). However, ACE inhibitors are not evaluated for cardio protective ability with lower doses of radiation (10 Gy or lower). Similarly, the drug *Simvastatin*, a lipid-lowering medication for lowering cholesterol has been observed to be capable of decreasing the radiation-associated injury to rats (88). However, critical data is lacking for understanding the ability of Simvastatin to mitigate cardiac damage following radiation (89). The plant polyphenol curcumin has been shown to have a potent anti-inflammatory and antioxidant properties (90).

Cardiac muscle toxicity can result in a concomitant loss of cardiac muscle and deterioration of the vasculature, ultimately resulting in cardiac failure. Current heart failure care can alleviate symptoms but cardiac myocytes that are killed during cancer therapies cannot be replaced or regenerated with current pharmaceuticals administered to-date. In light of the fact that most pharmaceutical interventions have not yet been demonstrated to be effective to repair cardiac damage, there arises a need for early detection of cardiac toxicity (91) and development of a new generation of therapeutics that are better able to more effectively prevent the cardiac injury caused by existing cancer therapeutics (92).

Cell based therapy to prevent RT-related cardiac injury—it has been investigated as a possible future treatment strategy for heart failure patients. Co-culturing stem cells with primary cells *in vitro* followed by injecting *in vivo* have demonstrated the ability of stem cells to engraft and differentiate into cells of cardiac nature. Myocytes isolated from cardiac tissue of rats have been shown as capable of inducing cardio- myogenic differentiation of endothelial progenitor cells (93, 94) and mesenchymal stem cells (95, 96). Mesenchymal stem cells injected into hearts of pig (97) or sheep (98) following myocardial infarction, have been shown to engraft long-term, express muscle-specific proteins as well as cells of vascular and smooth muscle origin (98). Despite the expression of cardiac proteins which are good indicators of cardiac differentiation, data is lacking for the stem cell's ability for differentiating into heart cells *in vivo*, alluding to the fact that merely injecting stem cells into heart may not be the best approach for cardiac muscle regeneration.

Activating stem cells residing within the heart may hold more promise as a therapeutic intervention strategy for heart regeneration. Scientific data exists for the ability of resident cardiac stem cells toward differentiating into the cardiac lineage. More specifically, the percentage of this population of dividing cardiac stem cells are shown to be increased in hearts undergoing acute infarction and those with end-stage cardiomyopathy when compared with normal cardiac tissue. Additionally, these cardiac stem cells display an increased commitment toward differentiation to the cardiac myocyte, smooth muscle and endothelial cell lineages within the infarcted and end-stage hearts as compared to hearts without abnormality or disease (99–101). Ongoing research is currently aimed at this differentiation process for understanding how to selectively increase the population of cells capable of regeneration which have highly sought after value for their functionality. Therefore, perhaps the best cell source for heart muscle regeneration is most likely the resident, cardiac stem cells if the proportion that becomes a thriving functioning heart cells could be enhanced. Further studies are needed to develop the cell based therapy specially targeted RT-induced cardiac injury.

## Conclusions

From various studies reviewed for this publication, it is evident that age at first radiation exposure plays a prominent role in cardiovascular related damage. The younger the age at first treatment, the greater the protection of the heart tissue and hence the lower is the risk. On the other hand, the older the age at first treatment the risk is significantly higher and the repercussions onset at an earlier time. It is also noted that by age 45–50 years, the risk of cardiovascular related damage risk increases by about 50%. This is of significant importance for general public and further studies and assessment by sex and treated conditions are to be published at a later time. We recommend more comprehensive long-term studies to be considered and evaluated as a function of time (up to ten years and beyond), sex (M/F), and radiation dose and type administered for various target sites.

A new class of radiation treatment procedures with particle therapy will be of greater challenge ahead in the years to come. At a rapid pace, nearly 20,000 patients per year during recent five years with ion therapy (protons and carbon) pose a potential challenge of cardio toxicity studies in near future. It is essential to establish the radiation related toxicity to the heart from particle therapy; it is believed that particle therapy is a rapidly growing approach for most cancer treatment protocols around the world.

Very likely it would be desirable for oncology research to encourage both medical and scientific explorations within the cardiac care and research communities to extend their follow-up for a greater period of time to discern any unforeseen cardiac complications which at present are most likely under-reported. Nonetheless, current radiation protocols far surpass the previous regimens in providing more radioprotection to critical organs including the heart. Much of the radiation related cardiotoxicity is associated with the use of traditional radiation approaches and older methods whereas the advanced modern therapies including particle therapy might reduce the immediate cardiac damage drastically. Advanced particle radiotherapy holds the promise for moving forward toward enhancing the efficacy of tumor cell killing and lowering the risk of cardiac complications from traditional radiation treatment approaches.

## Author contributions

All authors listed have made a substantial, direct and intellectual contribution to the work, and approved it for publication.

### Conflict of interest statement

The authors declare that the research was conducted in the absence of any commercial or financial relationships that could be construed as a potential conflict of interest.
